# *DYRK1A*, a Dosage-Sensitive Gene Involved in Neurodevelopmental Disorders, Is a Target for Drug Development in Down Syndrome

**DOI:** 10.3389/fnbeh.2016.00104

**Published:** 2016-06-03

**Authors:** Arnaud Duchon, Yann Herault

**Affiliations:** ^1^Department of Translational Medicine and Neurogenetics, Institut de Génétique et de Biologie Moléculaire et CellulaireIllkirch, France; ^2^UMR7104, Centre National de la Recherche ScientifiqueIllkirch, France; ^3^U964, Institut National de la Santé et de la Recherche MédicaleIllkirch, France; ^4^Université de StrasbourgIllkirch, France; ^5^PHENOMIN, Institut Clinique de la Souris, Groupement d’Intérêt Économique-Centre Européen de Recherche en Biologie et en Médecine, CNRS, INSERMIllkirch-Graffenstaden, France

**Keywords:** trisomy 21, neurodevelopmental disorder, mouse model, cognition, learning and memory, clinical trial, DYRK1A and kinase inhibitors

## Abstract

Down syndrome (DS) is one of the leading causes of intellectual disability, and patients with DS face various health issues, including learning and memory deficits, congenital heart disease, Alzheimer’s disease (AD), leukemia, and cancer, leading to huge medical and social costs. Remarkable advances on DS research have been made in improving cognitive function in mouse models for future therapeutic approaches in patients. Among the different approaches, DYRK1A inhibitors have emerged as promising therapeutics to reduce DS cognitive deficits. DYRK1A is a dual-specificity kinase that is overexpressed in DS and plays a key role in neurogenesis, outgrowth of axons and dendrites, neuronal trafficking and aging. Its pivotal role in the DS phenotype makes it a prime target for the development of therapeutics. Recently, disruption of *DYRK1A* has been found in Autosomal Dominant Mental Retardation 7 (MRD7), resulting in severe mental deficiency. Recent advances in the development of kinase inhibitors are expected, in the near future, to remove DS from the list of incurable diseases, providing certain conditions such as drug dosage and correct timing for the optimum long-term treatment. In addition the exact molecular and cellular mechanisms that are targeted by the inhibition of DYRK1A are still to be discovered.

## Introduction

Since Down (1866) described patients with mental retardation and characteristic faces, Down Syndrome (DS) has been recognized as one of the most common genetic disorders leading to intellectual disability. DS results from the presence of an extra copy of all or part of chromosome 21 (Lejeune et al., 1959). The clinical presentation of DS is complex and variable. A few features occur to some degree in every individual with trisomy 21, including 100% of patients with intellectual disability, hypotonia, and cranio-facial dysmorphology, 75% with brachycephaly or 60% with epicanthic fold, 40% with congenital heart disease, and an increased incidence of leukemia in DS that is 10- to 20-fold higher than that in the general population (Antonarakis et al., 2004). The most disabling phenotype for patients is the impaired intellectual and adaptive functioning, strongly contributed by defects in hippocampal- and prefrontal cortex-dependent functions (Pennington et al., 2003; Dierssen et al., 2009; Contestabile et al., 2010; Lott and Dierssen, 2010). These deficits are associated with both learning and short-term and long-term memory, resulting in a delay in cognitive development (Nelson et al., 2005) along with various aspects of language acquisition and comprehension (Chapman and Hesketh, 2000; Abbeduto et al., 2007). In the last decades, with better care and medical follow-up, the life expectancy of people with DS is increasing and is quickly approaching 60 years (Puri et al., 1995; Covelli et al., 2016). However, because DS patients show accelerated aging, including early onset dementia similar to Alzheimer’s disease (AD) (Zigman, 2013), and the increase in life expectancy does not follow the one observed in the general population or other groups with intellectual disability (Coppus, 2013).

Down syndrome features have largely been attributed to the overexpression of specific trisomic genes (Antonarakis et al., 2004), even if non-coding element (Elton et al., 2010) or DNA methylation (Lu and Sheen, 2013) could have significant effect on DS phenotypes. Transcriptome analyses showed that between 29 and 62% of trisomic genes are overexpressed by a factor of 1.5 with some variability and depending on the cell type (Saran et al., 2003; Aït Yahya-Graison et al., 2007; Prandini et al., 2007; Sultan et al., 2007; Laffaire et al., 2009; Moldrich et al., 2009) and that trisomy has an impact on large number of genes located in domains all over the genome (Letourneau et al., 2014). A few candidate genes have been selected for therapeutic approaches because of their brain related functions, their specific pattern of expression/localization and/or their contribution to signaling pathways involved in cognitive functions. In parallel, investigation of the relationship between phenotype and genotype using a panel of rare DS patients with only partial duplication of the chromosome 21 segment led to the hypothesis of the DS chromosomal region (DCR), in which a small set of genes (between D21S55-MX1) plays a major role in the determination of DS phenotypes including the development of cognitive disabilities (McCormick et al., 1989; Rahmani et al., 1989; Korenberg, 1990; Delabar et al., 1993; Sinet et al., 1994; Korbel et al., 2009; Lyle et al., 2009). Among the 33 genes in the DCR, dual-specificity tyrosine phosphorylation-regulated kinase 1A (*DYRK1A*), which has been always found overexpressed in DS patients and mouse models (Guimera et al., 1999; Dowjat et al., 2007), has received considerable attention because of its involvement in brain functions and processes that are altered in DS, and in the early onset of neurofibrillary degeneration, β-amyloidosis, neuronal loss and AD-like phenotypes in DS (Liu et al., 2008; Wegiel et al., 2011).

## DYRK1A Is A Kinase Involved in Neurodevelopmental Process and Brain Function

*DYRK1A* is the homologue of the *Drosophila* minibrain (*mnb*) which was named from the description of the brain phenotype observed in hypomorphic mutant flies (Tejedor et al., 1995). The DYRK family includes four additional mammalian subtypes including DYRK1B, DYRK2, DYRK3, and DYRK4. DYRK proteins show little sequence homology with other kinases outside of their catalytic domains but are highly conserved across species (Becker et al., 1998). DYRK1A can catalyze its own activation through auto-phosphorylation of a single tyrosine residue in its activation loop (Lochhead et al., 2005; Soundararajan et al., 2013; Soppa and Becker, 2015).

DYRK1A is expressed during embryonic neurogenesis. First, DYRK1A is transiently detected in preneurogenic region during early mouse embryogenesis from 8 to 10.5 days postcoitum (dpc). Then *Dyrk1a* expression was observed in cycling neuronal progenitor cells of the ventricular and subventricular zones at 14.5 dpc (Hammerle et al., 2008). The authors proposed that DYRK1A controls the mouse neuronal precursor exit from differentiation, leaving the cells in a quiescent state ready to differentiate while its expression is reduced (Hammerle et al., 2011). Finally, DYRK1A is expressed and translocated from the cytoplasm to the nucleus while the dendritic tree differentiated independently in several neuronal populations (Hammerle et al., 2003, 2008). In the adult mouse, the expression of *Dyrk1a* is found in several brain regions both in the cytoplasm and the nucleus (Marti et al., 2003).

The majority of the DYRK1A protein (almost 80%) is found associated with the cytoskeletal fraction in human and mouse brain, and the remaining protein is located in the cytosolic and nuclear fractions (Marti et al., 2003; Kaczmarski et al., 2014). The phosphorylated forms of DYRK1A are specific to subcellular localization in human and mouse brain. With only one residue phosphorylated (the conserved autophosphorylation site Y321) in the cytosolic DYRK1A and multiple heterogeneous phosphorylated sites found in the cytoskeletal and nuclear DYRK1A (Kaczmarski et al., 2014). Thus the function of DYRK1A could be regulated by the action of specific kinase(s) that will influence its stability or its ability to localize to nuclear, cytosolic or cytoskeletal compartments and thus to interact with specific substrates. Indeed the nuclear accumulation of DYRK2 is controlled by the “ataxia telangiectasia mutated” (ATM) dependent phosphorylation. When phosphorylated by ATM, DYRK2 dissociates from MDM2 (“transformed mouse 3T3 cell double minute 2”) and is no more degraded in the nucleus through a MDM2-dependent ubiquitination and thus could accumulate (Taira et al., 2010). This finding affecting DYRK localisation raises several questions such as whether a similar mechanism exists for DYRK1A and how it will be perturbed if there is an overdosage of the protein.

DYRK1A phosphorylates different targets depending upon its cellular localization (Marti et al., 2003; Park et al., 2009; Kaczmarski et al., 2014). It acts on a multitude of exogenous protein substrates, including transcription factors [CREB, NFAT (nuclear factor of activated T-cells), STAT3, FKHR, GLI1, RNApol2], splicing factors (cyclin L2, SF2, SF3), a translation factor (eIF2Be), miscellaneous proteins (glycogen synthase, caspase-9, Notch) or cytoskeletal target (TAU and MAP1B) and synaptic proteins (dynamin I, amphiphysin I, synaptojanin I; **Table 1**). Several targets listed here, might contribute to the role of DYRK1A in neuronal synaptic plasticity (Wegiel et al., 2004; Aranda et al., 2008; Murakami et al., 2009, 2012). Recent studies in Cos7 cells suggest that DYRK1A is involved in the regulation of dendritic spine formation through Neural Wiskott–Aldrich syndrome protein phosphorylation (Park et al., 2012). At the synaptic level, DYRK1A could regulate synaptic vesicle endocytosis via phosphorylation of AP180, dynamin I, amphiphysin I, and synaptojanin I, as demonstrated in isolated rat brain clathrin coated vesicle (Murakami et al., 2009, 2012). Clathrin-mediated endocytosis is essential for the recycling of membrane after neurotransmitter release (Saheki and De Camilli, 2012). DYRK1A is found in the pre-synaptic compartment of the neuromuscular synapse (Arque et al., 2013). Conversely, in the Drosophila neuromuscular junction, MNB acts as a synaptic kinase that promotes efficient synaptic vesicle recycling (Chen C.K. et al., 2014). Moreover, DYRK1A phosphorylation of GRIN2A modifies the biophysical properties of GRIN1/GRIN2A, two subunits of *N*-methyl-D-aspartate receptors (NMDAR) and controls NMDAR activity in neurons, which are involved in neural development, survival, synaptic plasticity and memory processes (Grau et al., 2014).

**Table 1:** Proteins that interact with MNB/DYRK1A.

Symbol	Name	Subcellular location	Biological process	Mouse Protein identification	Int.	Reference
Amph	Amphiphysin	Cytoplasm	Synaptic vesicle endocytosis	Q7TQF7	P	Murakami et al., 2006
App, b-amyloid	Amyloid precursor protein, β-amyloid precursor protein	Cytoplasm	Apoptosis, Cell adhesion, Endocytosis	P12023	P, RE	Kimura et al., 2007; Ryoo et al., 2008
Arip4	Androgen Receptor Interacting Protein 4	Nucleus	ATP, DNA and Nucleotide-binding	Q99NG0	Int.	Sitz et al., 2004
Braf	Braf transforming gene	Cytoplasm, Nucleus	ATP, Metal and Nucleotide-binding	P28028	Int.	Kelly and Rahmani, 2005
Casp-9	Cystein aspartyl protease Caspase 9	Cytoplasm, Nucleus	Apoptosis	Q8C3Q9	P	Laguna et al., 2008; Seifert et al., 2008
Ccnd1	Cyclin D1, cyclin family	Nucleus, Cytoplasm	Cell cycle and division, Transcription	P25322	P	Chen et al., 2013; Najas et al., 2015
Ccnl2	Cyclin L2, cyclin family	Nucleus	Transcription regulation	Q9JJA7	P	De Graaf et al., 2004
CreB	cAMP response element-binding protein	Nucleus	Differentiation, Transcription regulation	Q01147	P	Yang et al., 2001
Cry2	Cryptochrome Circadian Clock 2	Cytoplasm, Nucleus	Biological rhythms, Sensory transduction, Transcription regulation	Q9R194	P	Kurabayashi et al., 2010
CTD RnaP II	CTD of the RNA polymerase II	Nucleus	Transcription	P08775	P, DNA binding	Di Vona et al., 2015
Dnm1	Dynamin 1	Cytoplasm	Endocytosis	P39053	P	Chen-Hwang et al., 2002
Endophilin 1	Endophilin 1	Cytoplasm, Membrane	Endocytosis	Q62420	B	Murakami et al., 2009
Fkhr	Forkhead box O1	Cytoplasm, Nucleus	Apoptosis, Autophagy, Differentiation, Transcription regulation	Q9R1E0	P	Woods et al., 2001
Gli1	Glioma-associated oncogene 1	Cytoplasm, Nucleus	Differentiation, Transcription regulation	P47806	P	Mao et al., 2002
Grb2	Growth factor receptor bound protein 2	Cytoplasm, Nucleus	Cell differentiation	Q60631	Int.	Abekhoukh et al., 2013
Gsk3B	Glycogen synthase kinase 3 beta	Nucleus, Cytoplasm, Membrane	Differentiation, Neurogenesis	Q9WV60	P	Skurat and Dietrich, 2004
Grin2a	glutamate receptor, ionotropic, NMDA2A	Cell membrane, Cell junction	Ion transport, Transport	P35436	P	Grau et al., 2014
Hip1	Huntingtin interacting protein 1	Nucleus	Apoptosis, Differentiation, Endocytosis, Transcription regulation	Q8VD75	P	Kang et al., 2005
Kip1	Cyclin-dependent kinase inhibitor 1B	Cytoplasm	Cell cycle	P46414	P	Soppa et al., 2014
Lin52	Protein lin-52 homolog	Nucleus	Cell cycle, transcription	Q8CD94	P	Litovchick et al., 2011
Map1b	Microtubule-associated protein	Cytoplasm	Axon extension, intracellular transport	P14873	P	Scales et al., 2009
Mek1	Dual specificity mitogen-activated protein kinase kinase 1	Cytoplasm, Nucleus	ATP-binding, Nucleotide-binding	P31938	Int.	Kelly and Rahmani, 2005
Nfatc	Nuclear factor of activated T cells	Cytoplasm, Nucleus	Transcription regulation	O88942, Q60591, P97305, Q8K120	P	Arron et al., 2006; Gwack et al., 2006
Notch	Notch Signaling Pathway	Nucleus	Angiogenesis, Differentiation, Transcription	Q01705	P	Fernandez-Martinez et al., 2009
Nrsf / Rest	RE1-silencing transcription factor	Nucleus	Transcription regulation	Q8VIG1	RE	Canzonetta et al., 2008
P53	Transformation related protein 53	Cytoplasm, Nucleus	Transcription Apoptosis, Cell cycle, Necrosis, Transcription	P02340	P	Park et al., 2010
Phyhip	Phytanoyl-COA Hydroxylase-interacting protein	Cytoplasm	Activation of mitophagy	Q8K0S0	B	Bescond and Rahmani, 2005
Park2	Parkin	Cytoplasm, Nucleus	Autophagy, Transcription regulation	Q9WVS6	P	Im and Chung, 2015
Psen1	Presenilin1	Cytoplasm	Apoptosis, Cell adhesion, Notch signaling pathway	P49769	P	Ryu et al., 2010
Ras	GTPase Ras	Cytoplasm	Cell proliferation	Q61411, P32883, P08556	Int.	Kelly and Rahmani, 2005
Rcan1/Dscr1	Regulator of calcineurin 1	Cytoplasm, Nucleus	Calcineurin-NFAT signaling cascade	Q9JHG6	P	Song et al., 2013
Srsf1	Serine/arginine-rich splicing factor 1	Cytoplasm, Nucleus	mRNA processing, splicing and transport	Q6PDM2	P	Shi et al., 2008
Srsf2	Serine/arginine-rich splicing factor 2	Nucleus	RNA splicing	Q62093	P	Qian et al., 2011
Sept4	Septine4	Cytoplasm	GTP-binding, Nucleotide-binding	P28661	P	Sitz et al., 2008
Sf3b1/Sap155	Splicing factor 3b, subunit 1	Nucleus	mRNA processing, mRNA splicing	Q99NB9	P	De Graaf et al., 2006
Sirt1	Sirtuin 1	Cytoplasm, Nucleus	Apoptosis, Differentiation, Myogenesis, Transcription	Q923E4	P	Guo et al., 2010
Snca	a-synuclein	Cytoplasm, Nucleus	Synaptic function	O55042	P	Kim E.J. et al., 2006
Snr1	Integrase interactor 1	Nucleus	Cell cycle, Neurogenesis, Tanscription regulation	Q9Z0H3	P	Kinstrie et al., 2006
Spry2	Sprouty2	Cytoplasm	Developmental protein	Q9QXV8	P	Aranda et al., 2008
Stat3	Signal transducer and activator of transcription 3	Cytoplasm, Nucleus	Acute phase, Transcription, Transcription regulation	P42227	P	Kurabayashi et al., 2015
Synj1	Synaptojanin 1	Cytoplasm	Endocytosis	Q8CHC4	P	Chen C. et al., 2014
Tau	Microtubule-associated protein Tau	Cytoplasm	Brain development	P10637	P	Ryoo et al., 2007
Wasl	Neural Wiskott-Aldrich syndrome protein	Cytoplasm, Nucleus	Cell cycle, Cell division, Mitosis, Transcription	Q91YD9	P	Park et al., 2012
Wdr68	DDB1 and CUL4 associated factor 7	Cytoplasm, Nucleus	Ubl conjugation pathway	P61963	B	Morita et al., 2006; Miyata and Nishida, 2011


*P, phosphorylation by Dyrk1A; RE, regulate expression; Int., interaction with Dyrk1A; B, binding with Dyrk1A.*

## Dyrk1A Dosage and Neurodevelopmental Diseases

**FIGURE 1:**
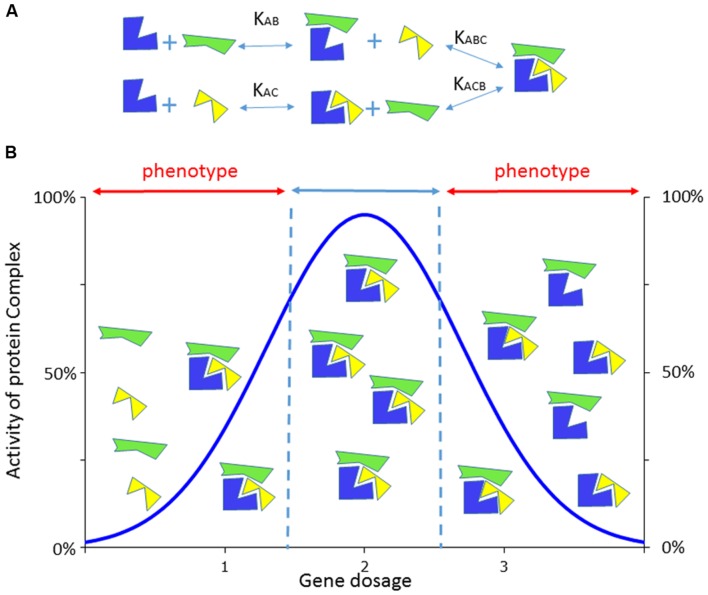
**Consequence of the dosage effect on the activity of a multiprotein complex.** **(A)** Example of a dosage sensitive gene whose encoded protein (in blue) is able to form a tripartite complex with two partners (in yellow or green), using different constant of association/dissociation. **(B)** The formation of the complex will be altered by the level of expression of the blue protein compared to the yellow or green ones which are not dosage dependent here. With two copies of the dosage sensitive genes, complexes will be formed with the expected 100% level of activity while in only one (or three copies) are expressed the corresponding complexes will be disrupted delivering only partial activity that would lead when below (or above) a certain threshold to phenotypes. Adapted from (Veitia et al., 2008).

*DYRK1A* is a paradigm of a dosage sensitive gene with its underexpression, caused by heterozygous disruption or loss-of-function mutations, leading to MRD7 and its overexpression contributing to DS cognitive dysfunction. Such a variation in gene dosage could have major impact on multiprotein complex at the level of enzymatic activities or transcriptional regulation (**Figure 1**) (Veitia et al., 2008). The first MRD7 cases were translocation disrupting DYRK1A in two patients with microcephaly, severe mental retardation without speech, anxious autistic behavior, or dysmorphic features (Møller et al., 2008). Then, a second study identified patients isolated from large screen of 3,009 intellectually disabled individuals who presented a *de novo* heterozygous deletion of the last three exons of *DYRK1A* (van Bon et al., 2011). Several reports showed heterozygous mutations in *DYRK1A* in patients with multiple phenotypes, including developmental delays or intellectual disability with autism spectrum disorder, microcephaly, epileptic seizures, facial dysmorphisms and cardiac defects (Courcet et al., 2012; O’Roak et al., 2012; Bronicki et al., 2015; Ruaud et al., 2015; Van Bon et al., 2015) defining a new syndromic condition (Courcet et al., 2012; Bronicki et al., 2015; Van Bon et al., 2015). Similar neuro-developmental phenotypes (delay in eyelid and ear opening, in the appearance of the righting reflex and of the Preyer’s reflex), microcephaly, locomotor activity and coordination (sensorimotor tets, balance, gait analysis, rotarod deficiency) cognition defects (delay in the startle response, spatial memory deficit in the Morris water maze and in the radial-arm water maze, memory recognition in the object recognition paradigm) and reduced dendritic tree of the layer III pyramidal cells were observed in mouse heterozygous knockout models (Fotaki et al., 2002; Benavides-Piccione et al., 2005; Dierssen and Martinez de Lagran, 2006; Arqué et al., 2008, 2009). Homozygous *Dyrk1a* knock-out (KO) mice die in utero with growth retardation, reduced body size and morphological developmental delay of the primitive organs. In an inbred genetic background, newborn heterozygous KO mice have reduced neonatal viability and decreased body size (Fotaki et al., 2002). Nevertheless, approximately 30% of the heterozygous KO mutants can survive in a mixed background. Their adult brains present increased neuronal densities in some brain regions and a specific decrease in the number of neurons in the superior colliculus, which exhibits a significant size reduction (Fotaki et al., 2002).

The role of DYRK1A in DS is deduced from several studies in mouse models. Mainly, mouse models overexpressing *Dyrk1a* or trisomic for genomic segments, homologous to Hsa21 and encompassing *Dyrk1a*, showed that *Dyrk1a* overdosage was sufficient to impair cognition with defects similar to DS people (For review see Ahn et al., 2006; Dierssen and Martinez de Lagran, 2006; Dierssen et al., 2009; Guedj et al., 2012; Herault et al., 2012). Several strategies to reduce the expression of *Dyrk1a* in DS mouse models have shown that *Dyrk1a* overdosage was necessary for DS related phenotypes. First, the normalization of *Dyrk1a* expression was achieved via adeno-associated viral delivery of a shRNA sequence specific for *Dyrk1a*. If injected in the hippocampal region, hippocampal-dependent defects in long term potentiation (LTP), a persistent increase in synaptic strength following high-frequency stimulation of a chemical synapse, and related memory performance (Morris water maze and fear conditioning) were restored in Ts65Dn mice (Altafaj et al., 2013). In addition, motor alterations were reduced after striatal injection of AAV-shRNA in transgenic mice overexpressing *Dyrk1a* (Ortiz-Abalia et al., 2008). These results demonstrated that normalization of *Dyrk1a* expression in the adult brain when neuro-developmental alterations have already occurred is sufficient to ameliorate synaptic plasticity changes via its effect on the Erk/CREB signaling pathway, that participates in the LTP induction (Thomas and Huganir, 2004), considered to be one of the major cellular mechanism sustaining learning and memory (Bliss and Collingridge, 1993; Cooke and Bliss, 2006). Second, the normalization of *Dyrk1a* copy number in trisomic mice was shown to improve hippocampal-dependent learning, presumably due to the recovery of synaptic plasticity, the enhancement of adult hippocampal cell proliferation and differentiation and/or the improvement of the balance between inhibitory and excitatory synaptic markers (Garcia-Cerro et al., 2014; Jiang et al., 2015). In addition, three copies of *Dyrk1a* was also shown to cause retinal structural and functional alterations in trisomic mice, and normalization of *Dyrk1a* copy number completely rescued both the morphological and functional visual phenotypes (Laguna et al., 2013).

Overall the phenotypes observed in MRD7 and DS affect similar areas and functions. Brain function is altered in both conditions but with different impact on intellectual abilities. As mentioned previously DS defects includes both short- and long-term memory with delay in learning and in language acquisition and comprehension (Chapman and Hesketh, 2000; Pennington et al., 2003; Nelson et al., 2005; Abbeduto et al., 2007; Dierssen et al., 2009; Contestabile et al., 2010; Lott and Dierssen, 2010) whereas in MRD7 autistic traits, repetitive behavior, feeding difficulties are found associated with more severe intellectual disabilities, speech delay or absence (Courcet et al., 2012; O’Roak et al., 2012; Bronicki et al., 2015; Ruaud et al., 2015). The vision is affected in both conditions but a more precise phenotypic characterization is needed in MRD7. Microcephaly is observed in MRD7 and in *Dyrk1a* heterozygous mice. Macrocephaly with more precise regional morphological changes are detected in transgenic mice overexpressing *Dyrk1a* alone (Guedj et al., 2012) but the macrocephaly is not observed in DS mouse models such as the Ts65Dn, suggesting that other genes are contributing to the DS phenotypes (Jiang et al., 2015). Accordingly DS people have a more reduced brain size, with a particular impact on the cerebellum (Pinter et al., 2001; Guihard-Costa et al., 2006), than the normal population. The microcephaly observed in MRD7 should be a direct consequence of abnormal neuronal progenitor proliferation and differentiation due to the loss-of-function of DYRK1A, and induce delay in brain development. On the opposite developmental changes do not seem to be causing the defects rescued by normalizing DYRK1A in DS models. Indeed treatment targeting DYRK1A in adult preclinical model demonstrate that the increase in DYRK1A gene dosage seems perturbed more the physiological function of DYRK1A even though the situation should be more complex in both diseases.

### DYRK1A Dosage Variation and Impact on Cellular and Molecular Mechanisms

Cognitive dysfunctions observed in MRD7 and in DS result from DYRK1A misdosage during in utero development or in the adult brain. As in MRD7, the DS brain volume is smaller (Guihard-Costa et al., 2006), and this difference persists at post-natal stages (Pinter et al., 2001). Development of the superior temporal neocortex is abnormal with defect in axonal and dendritic arborization (Becker et al., 1986; Golden and Hyman, 1994). Several studies conducted in humans and DS mouse models have suggested the presence of a defect in neurogenesis and increased cell death in the hippocampus. This defect is probably due to alterations of the cell cycle in neuronal progenitors (Contestabile et al., 2007; Esposito et al., 2008; Guidi et al., 2008).

A number of studies have been conducted and demonstrate that DYRK1A dosage is key role for neurogenesis and neuronal maturation (for review see Park et al., 2009; Tejedor and Haemmerle, 2011). DYRK1A regulates proliferation and neuronal differentiation through the phosphorylation of p27Kip1 and Cyclin D1 (CCND1) (Hammerle et al., 2011; Soppa et al., 2014). *Dyrk1a* gain of function experiments were shown to stop cell proliferation with an increased number of cells expressing p27Kip1, a cyclin dependent kinase inhibitor acting as a main negative regulator of the cell cycle of neurons. Conversely its loss-of-function triggered proliferation and cell death through a p27Kip1 downregulation (Hammerle et al., 2011). *Dyrk1a* transient expression control the neuronal precursor exit from differentiation through CCND1 and P27Kip1, leaving the cells in a quiescent state ready to differentiate while its expression reduced (Hammerle et al., 2011). DYRK1A can also regulate the G1-phase of the cell cycle of fibroblast cells through the direct phosphorylation of CCND1 (at T286), its nuclear export, degradation, and relative level with p21, all together determining the cycle entry versus exit decision. As such the G1 phase is extended in trisomic fibroblasts due to a lower CCND1 level that could be counteracted by inhibiting DYRK1A (Chen et al., 2013). The same mechanism has been found while DYRK1A controls the cell exit of ventricular neuronal progenitor cells in mouse embryos. In this compartment, *Dyrk1a* overexpression, through in utero electroporation of mouse embryos, inhibits cell cycle progression and induces premature neuronal differentiation without affecting the capacity to migrate or to differentiate in the post-natal cortex (Yabut et al., 2010). Accordingly in transgenic *Dyrk1a* mouse embryos, the G1 phase is increased in progenitor cortical stem cells due to CCND1 phosphorylation by DYRK1A, producing a deficit in cortical projection neurons that persists in postnatal stages (Najas et al., 2015). This phenotype is also present in the Ts65Dn DS mouse model which are trisomic for a large region homologous to the human chromosome 21.

Nevertheless additional mechanisms are also perturbed by change in DYRK1A dosage. DYRK1A interferes with the NFATc pathway that is critical for the regulation of vertebrate development, organogenesis and neuronal development (Graef et al., 2001; Nguyen and Di Giovanni, 2008). Overexpression of *Dyrk1a* acts synergistically with GSK3, as a priming kinase, to inhibit NFAT-dependent transcription in cortical neurons stimulated by FGF8 and heart valve elongation during development of the Ts1Cje DS models, through increase in NFAT nuclear export (Arron et al., 2006). Conversely DYRK1A and DYRK2 were found to phosphorylate NFAT regulatory domain in Drosophila (Gwack et al., 2006). As expected overexpression of both *Dyrk1a* and *Rcan1* results in delayed differentiation of neuronal progenitor cells, with a marked cell-cycle re-entry and alteration of laminar positioning. This phenomenon leads to a reduction of nuclear NFAT localization and can be rescued by expressing a constitutively active form of NFAT (Kurabayashi and Sanada, 2013). As expected interfering with *Rcan1* or *Dyrk1a* overdosage in the Ts1Cje models, or expressing the same constitutive active form of NFAT, rescue the differentiation process, found affected in the trisomic mice. As such the deregulation of the DYRK1A/RCAN1/NFAT pathway leads to developmental alterations which should impact brain size and neuronal density, two traits altered in DS.

In addition, the aberrant enhancement of astrocytic differentiation of cortical progenitor cells has been described in the Ts1Cje mouse model. It is promoted by DYRK1A acting on STAT3, a transcription factor critical for astrogliogenesis (Kurabayashi et al., 2015). This defect could be related to phenotypes observed in DS astroglia. Indeed, differentiation of human induced Pluripotent Stem cells (hiPSc)-from DS patients revealed defect in the neuronal maturation, synapse formation and neurogenesis due to specific overexpression of S100B in medium derived from hIPSc-derived astrocytes (Chen C. et al., 2014). Unfortunately, no S100b-dependent and specific phenotypes have been found so far in DS trisomic models (Yu et al., 2010a,b; Duchon et al., 2011; Belichenko et al., 2015; Jiang et al., 2015) (Duchon and Herault, personal communication) but further analysis in older individuals or to address the function of the tripartite synapse (Pereira and Furlan, 2010) might unravel additional changes.

Overall the variation in DYRK1A activity may impact the proliferation of progenitors cells, the function of the REST complex, leading to the microcephaly observed in MRD7 while in DS premature differentiation will lead to a reduced pool of mature neurons. Presumably both mechanism will contribute to the decreased brain size observed in DS, and cell density alterations found in animal models.

DYRK1A controls neuronal morphogenesis by regulating cytoskeletal dynamics, and overexpression of *Dyrk1a* in mice is sufficient to recapitulate the dendritic alterations observed in DS patients (Martinez de Lagran et al., 2012). In the adult, connectivity and plasticity are also impacted at different levels. In trisomic mice, dendritic arbor size of neocortical pyramidal cells is smaller, and the peak branching in the arbor was less complex (Dierssen et al., 2003). Total synapse density and synapse-to-neuron ratios are significantly lower in trisomic context (Kurt et al., 2004). Pre-synaptic and post-synaptic elements are significantly enlarged in the hippocampus, the motor and somatosensory cortex, the entorhinal cortex, and the medial septum (Belichenko et al., 2004). Moreover, there is a significant alteration of inhibitory synapses in the fascia dentata (Belichenko et al., 2009). These defects are probably responsible of changes in LTP and long-term depression (LTD), which have been observed in DS mouse models (Siarey et al., 1997, 1999, 2005; Kleschevnikov et al., 2004; Belichenko et al., 2007; Fernandez et al., 2007). Among other possible mechanisms, reduced *N*-methyl-D-aspartate (NMDA) receptor activation may contribute with an excessive GABAergic inhibition (Kleschevnikov et al., 2004; Costa and Grybko, 2005; Belichenko et al., 2007). In addition increase in GABAergic neurons and changes in the excitatory/inhibitory balance are observed in DS models (Altafaj et al., 2008; Grau et al., 2014; Souchet et al., 2014). Both observations are important but nothing is known at present linking DYRK1A to the glutamatergic synapse or to the GABAergic neurons.

Finally, in the later stages of life, DS patients present a neuropathology similar to AD that will evolve in dementia for 80% of patients after the age of 65 (Strydom et al., 2010; McCarron et al., 2014). This degenerative modification appears in conjunction with the presence of Aβ and tau lesions in several brain regions (Wisniewski et al., 1985). Additionally, a deficit in cholinergic neurons similar to the deficit that occurs in AD has been observed in DS during aging (Contestabile and Ciani, 2008). Even if there is a high prevalence of dementia, not every DS patient develops the accompanying clinical symptoms (Nieuwenhuis-Mark, 2009). Interestingly, overexpression of DYRK1A and some direct targets, such as APP and MAPT, contributes to the early onset of neurofibrillary degeneration, β-amyloidosis, neuronal loss and dementia in DS (Rovelet-Lecrux et al., 2006, 2010; Salehi et al., 2006; Liu et al., 2008; Wegiel et al., 2011; Rovelet-Lecrux and Campion, 2012; Park and Chung, 2013).

DYRK1A is expressed in the nucleus and can also control the expression and interact transcription factor such as the RE1-silencing transcription factor/neuron-restrictive silencer factor REST/NRSF–SWI/SNF chromatin remodeling complex (Canzonetta et al., 2008). Through these mechanisms, increase of *Dyrk1a* gene dosage induces a SWI/SNF-linked deregulation of gene clusters involved in the neuronal phenotypic traits of DS (Lepagnol-Bestel et al., 2009). *Dyrk1a* overexpression also leads to alteration in the transcriptome, and these changes affect the NMDA type glutamate receptor with an alteration of NMDA-induced calcium dynamics (Grau et al., 2014). More recently DYRK1A has been showed to act as a transcription factor, phosphorylating the carboxy terminal domain of the RNA polymerase 2 in HeLa cells (Di Vona et al., 2015). Nevertheless no evidence have been found yet for an impact of DYRK1A dosage on the direct interaction of DYRK1A with the RNApol 2 in MRD7 or DS models.

## An Eruption of Dyrk1A Kinase Inhibitors

Inhibition of DYRK1A activity represents a new field of study, and a growing number of active molecules to target this protein have been isolated over the last 5–10 years (for review see Becker and Sippl, 2011; Ionescu et al., 2012; Smith et al., 2012; Tell and Hilgeroth, 2013; Becker et al., 2014; Abbassi et al., 2015).

Historically, several compounds were isolated that exhibit dual inhibition of both DYRK family members and cell division cycle-like kinase (CLK family kinases). Effectively, the CLK and DYRK kinase families are part of the CMGC group of the eukaryotic kinome; this group, named after the initials of some members, exhibits auto-phosphorylation activity in addition to phosphorylating serine and tyrosine residues of specific substrates (Aranda et al., 2011). The CLK family has been targeted for the regulation of alternative splicing. CLK family kinases are involved in controlling HIV-1 gene expression (Wong et al., 2011), hepatic gluconeogenesis (Rodgers et al., 2010), cancer (Liu et al., 2013) and neurodegenerative disease such as AD, and their inhibition can be used as a therapeutic strategy (Jain et al., 2014). Thus, many inhibitors have been developed in recent years and are still under investigation for the treatment of DS.

Several compounds with inhibitory activity were originally isolated from natural sources. For example, harmine, a β-carboline alkaloid with a pyrido[3,4-b]indole ring structure was first isolated from the South American vine *Banisteriopsis caapi*. This molecule has been shown to be an ATP-competitive inhibitor of DYRK1A *in vitro*, with less potency against other DYRK family members (Laguna et al., 2008; Gockler et al., 2009; Adayev et al., 2011). Tg(Dyrk1a) mice, which overexpress *Dyrk1a* like people with DS, treated with harmine showed a significant decrease of homocysteine and liver ERK1/2 phosphorylation (Noll et al., 2012). Additionally, harmine prevents premature maturation of neuronal progenitors isolated from Ts65Dn (Mazur-Kolecka et al., 2012). Finally, harmine or its derivatives have been shown to reduce the levels of multiple phosphorylated forms of tau protein in tau overexpressing H4 neuroglioma cells, which are important in the pathological progression of AD (Frost et al., 2011). However, β-carboline analogs possess additional properties such as the inhibition of monoamine oxidase A, a target for depression (Kim et al., 1997), but with significant drawbacks, such as their hallucinogenic properties and a plethora of psychoactive effects, which limit their use *in vivo* (Fuentes and Longo, 1971). The flavonoid epigallocatechin-3-gallate (EGCG) was the second compound used but the first to be shown to improve cognition in DS models and in humans. Flavanoids, are characterized by having a benzopyrane skeleton, with a pyrane ring bearing at least one aromatic ring. EGCG is also a natural polyphenol and is a major catechin component of green tea leaves (*Camellia sinensis*) and was identified as a non-competitive ATP inhibitor of DYRK1A (Bain et al., 2003; Adayev et al., 2006). Both EGCG and harmine can fully restore the endocytic defects of hippocampal neurons in mouse models that overexpress *Dyrk1a* (Kim et al., 2010). In human DS- iPSCs, DYRK1A inhibition by EGCG treatment during neural induction and neuronal differentiation induces an improvement in the number of neurons and promotes dendritic development (Hibaoui et al., 2014). *In vivo* and *in vitro* studies of DS mouse models treated with a green tea extract or EGCG extract demonstrated an improvement in brain structure, adult neurogenesis, synaptic plasticity and learning and memory (Xie et al., 2008; Guedj et al., 2009; Pons-Espinal et al., 2013; De la Torre et al., 2014). EGCG also regulates the expression of REST, which is a modulator of genes that encode fundamental neuronal functions, via inhibition of DYRK1A in embryonic stem cells and in the mouse cortex (Canzonetta et al., 2008). Moreover, a pilot study of EGCG in young adults with DS demonstrated effects on memory recognition, working memory and quality of life (De la Torre et al., 2014). EGCG has been demonstrated to be a safe substance after repeated administration in humans and can be easily found as a dietary supplement because of its anti-oxidant properties (Kanwar et al., 2012). A second trial (phase II) has already been conducted by Dierssen and coworkers, and the results are highly anticipated. However, some problems with this compound remain, such as its complex pharmacokinetic properties, poor bioavailability, multiple and heterogeneous effects on signaling pathways and the degree of purity of the commercially available compound (Lambert et al., 2007; Kanwar et al., 2012; Lorenz, 2013). In addition, EGCG was found to have a low inhibitory effect on cannabinoid receptor 1 (CNR1) activity (Korte et al., 2010), which could have negative consequences for long-term treatment as the well-known rimonabant, an inverse agonist directing CNR1. Rimonabant was used as an anorectic and antiobesity drug and was removed from the market after reports of severe depression and suicide in treated people (Thomas et al., 2014). Thus, it is essential to concentrate future efforts on finding a more specific DYRK1A inhibitor, with no interference with CNR1.

Other inhibitors were isolated from natural sources. This is the case for several marine alkaloids, such as variolins, a family containing a central pyrido[3′,2′:4,5]pyrrolo[1,2-c] pyrimidine core substituted with a 2-aminopyrimidine ring and meridianins, and a family of 3-(2-amino-pyrimidine)indoles (Gompel et al., 2004; Giraud et al., 2011; Tahtouh et al., 2012) as well as meriolins, which are a chemical hybrid between the natural products meridianins and variolins (Echalier et al., 2008). Olomoucine is one of the first CDK inhibitors to be developed, and two of its derivatives, family members of 2,6,9-trisubstituted purines roscovitine and purvalanol, also act on DYRK1A (Vesely et al., 1994; Gray et al., 1999; Bain et al., 2003). Staurosporine belongs to the class of indolocarbazoles, which bear a single sugar residue bound to both indole nitrogens, and was originally isolated from the bacterium *Streptomyces stauroporus*. This compound has exhibited potential as an inhibitor of DYRK1A, but its inhibitory activity was not selective enough among a large array of kinases (Sanchez et al., 2009).

Many inhibitors of DYRK1A kinase activity that have been developed so far are type I inhibitors; i.e., they target the ATP binding site of the kinase in its active conformation when the activation loop is phosphorylated. They have a chemical structure close to the adenine nucleus like many nitrogen heterocyclic compounds such as quinoline, quinazolines, pyrimidines, pyrrolopyrimidines, pyrrolopyridines, and pyrazolopyrimidines. These heteroaromatic cores are often capable of highly efficient binding to proteins because of their shape and hydrophobic nature. Their advantages are their lack of flexibility combined with hydrogen bonding potential from their heteroatoms that can provide a level of target selectivity. Additionally, a rapid exploration of the effect of adding different substituents is facilitated by the applicability of parallelizable reactions, and finally, two or more substitution positions can be explored without the complication of introducing a stereocenter. Their major disadvantages are their hydrophobic nature and flat shape, resulting in low aqueous solubility (Pitt et al., 2009). These inhibitors have higher potency on substrate phosphorylation than on autophosphorylation. Serine and threonine phosphorylation of substrates is inhibited with lower impact on autophosphorylation and the remaining tyrosine kinase activity. Such a difference may reflect the accessibility of the inhibitor target site. DYRK1A kinase activity is closely regulated, with two successive conformational states proposed: the first immature state allows the enzyme to act on the tyrosine residue, while the second more mature state, being irreversible, reacts with the serine/threonine residue (R(X_1-2_)S/TP) (Lochhead et al., 2005). The two-states model was enriched with the two conformations having slight differences in reacting with tyrosine or serine/threonine, with the immature state having lower catalytic activity and different equilibrium for the amino acid than the mature state (Walte et al., 2013). Additionally, several inhibitors have been synthesized, such as Tg003 (Muraki et al., 2004) and INDY (Ogawa et al., 2010) two benzothiazole derivatives. For example, DANDY, a new 3,5-diaryl-7-azaindole that demonstrates potent inhibition against DYRK1A kinase activity (Gourdain et al., 2013). HCD160 and its derivatives (Kim N.D. et al., 2006; Koo et al., 2009), a series of substituted 6-arylquinazolin-4-amines (Mott et al., 2009; Rosenthal et al., 2011), a new 3-(6-hydroxyindol-2-yl)-5-(phenyl)pyridine (Kassis et al., 2011), a series of aryl-substituted aminopyrimidines (Coombs et al., 2013), a new 7-substituted pyrido[2′,3′:4,5]furo[3,2-d]pyrimidin-4-amines (Deau et al., 2013), an 8-arylpyrido[30′20′:4,5]thieno[3,2-d]pyrimidin-4-amines (Loidreau et al., 2015), and a series of hydroxybenzothiophene ketones (Smith et al., 2012; Schmitt et al., 2014). To date, no DYRK-specific or CLK-specific inhibitors have been reported, certainly because there is a high degree of conservation of the ATP binding site inside the CLK and DYRK kinase families. Nevertheless novel interesting compounds are in development (Rüben et al., 2015). Even if some cellular side effects have been reported for several of the listed compounds, none of them have yet been tested in DS models. Moreover, the activity of these compounds is usually stronger on CLK kinases, excluding varioline B, leucettine 41 and harmine (Tahtouh et al., 2012).

Recent progress on more specific and selective inhibitor has already been made. For example, we note the development of a new bioluminescent reporter assay for evaluation of DYRK1A inhibitors. This system led to the identification of (*Z*)-5-[(2,3-dihydrobenzofuran-5-yl)methylene]-2-iminothiazolidin-4-one (referred to as CaNDY: CDC37 association inhibitor for DYRK1A) as a strong inhibitor of DYRK family kinases. In addition to inhibition potential, CaNDY decreases DYRK1A molecules in cells, thus efficiently suppressing DYRK1A activity compared to simple inhibition of kinase activity (Sonamoto et al., 2015). Substituted quinazolines are a common pharmacophore for ATP-competitive kinase inhibitors, and the 5 novel thiazolo[5,4-f]quinazoline derivatives that have been synthesized (EHT 5372, 6840, 1610, 9851 and 3356) are among the most potent DYRK1A/1B inhibitors disclosed to date. In particular, they are more potent than NCGC-00189310 (Rosenthal et al., 2011) and leucettine L41 (Tahtouh et al., 2012), the two most active reference inhibitors tested during the screening campaign (Foucourt et al., 2014; Coutadeur et al., 2015). In addition, an indirubin derivative was inactive toward cyclin-dependent kinase 5 (CDK5), GSK3β, and casein kinase 1 (CK1) while exhibiting good selectivity and affinity for DYRK kinases (Myrianthopoulos et al., 2013). Consequently, the development of type II or III inhibitors, non-ATP-mimetics that are anchored to more diverse regions of the ATP binding site, may result in more selective inhibitors while also targeting the premature DYRK1A kinase. For example KHCB19 is a dichloroindolyl enaminonitrile derived from bauerine C, a β-carboline alkaloid originally isolated from the blue-green alga *Dichothrix baueriana*. This compound exhibits a unique ‘non-ATP mimetic like’ binding mode to CLK1 and also acts on DYRK1A (Fedorov et al., 2011). The non-ATP competitive inhibitors, called type II and type III inhibitors act by inducing a conformational shift in the target enzyme such that the kinase is no longer able to function. This unusual binding mode highlights the opportunity to develop very potent and specific inhibitors with new chemical profiles because they offer the possibility to overcome the major problem of the type I inhibitor (Garuti et al., 2010). Nevertheless even if a more potent and specific inhibitor would be better, too much inhibition may be deleterious as in the case of loss-of-function mutation of *DYRK1A* in MRD7. In addition DYRK1A specific inhibition on substrates phosphorylation versus autophosphorylation would be interesting to determine. A specific substrate kinase inhibition may have a more beneficial effect rather than targeting the autophosphorylation. The situation is even more complex considering that the target site of the inhibitor might have different accessibility depending on the two conformations of DYRK1A.

Modulation of DYRK1A in MRD7 by increasing its activity would be a good strategy to alleviate the severe cognitive deficits present in the disease, in particular for loss-of-function mutations. Nevertheless only a few proteins are known to increase DYRK1A activity (see **Table 2**) and no DYRK1A compound activator has been described to date. Thus further work is needed to investigate such a strategy for MRD7.

**Table 2:** Proteins that regulate Dyrk1A activity.

Symbol	Name	Subcellular location	Biological process	Mouse Protein identification	Interaction	Reference
14-3-3	14-3-3 proteins	Nucleus	Brain development	Q9CQV8, P62259, P61982, P68510, P68254, P63101, O70456	Binding	Kim et al., 2004; Alvarez et al., 2007
Fgfb	Basic fibroblast growth factor	Nucleus	Angiogenesis, Differentiation	P15655	Not described	Yang et al., 2001
E1A	Human adenovirus early region 1A	Nucleus	Oncoprotein		Protein Interaction	Zhang et al., 2001
E2f1	Transcription factor E2F1	Nucleus	Apoptosis, Cell cycle, Transcription	Q61501	Not described	Maenz et al., 2008
Lats2	Large tumor suppressor 2	Cytoplasm, Nucleus	Cell cycle, Cell division, Mitosis	Q7TSJ6	P	Tschop et al., 2011


## Conclusion

Major steps have been achieved to establish the role of DYRK1A in intellectual disabilities such as DS and MRD7 syndromic condition involving heterozygous loss-of-function mutations affecting DYRK1A. These two neurodevelopmental disorders share common features resulting from the alteration of DYRK1A-controlled mechanisms such as neuronal proliferation and differentiation. However, many questions on DYRK1A inhibitors remain unanswered. Are type 1 DYRK1A inhibitors blocking the ATP binding site better suited than type 2 or 3 inhibitors selective for a particular conformation of DYRK1A? What are the cellular targets of DYRK1A: GABAergic or glutamatergic neurons, glial cells?

Undeniably DYRK1A inhibitors represent a promising class of molecules to improve cognitive deficits in people with DS. The pilot clinical trials in adults treated with EGCG is promising, showing a modest gain of cognition (De la Torre et al., 2014). Perinatal treatment during earlier phases of brain development is attractive for improving synaptic plasticity, keeping in mind that DYK1A plays an important role in brain growth by controlling neuronal proliferation and differentiation. Additional studies will be needed to evaluate the use of DYRK1A inhibitors during perinatal periods.

## Author Contributions

All authors listed, have made substantial, direct and intellectual contribution to the work, and approved it for publication.

## Conflict of Interest Statement

The authors declare that the research was conducted in the absence of any commercial or financial relationships that could be construed as a potential conflict of interest.
